# Assessment of calf muscle constitution in chronic Achilles tendon disease using Dixon-based MRI

**DOI:** 10.1007/s00256-024-04845-7

**Published:** 2024-12-11

**Authors:** Sophia S. Goller, Georg W. Kajdi, Stephan Wirth, Jess G. Snedeker, Reto Sutter

**Affiliations:** 1 Department of Radiology, Balgrist University Hospital, Faculty of Medicine, University of Zurich, Forchstrasse 340, 8008 Zurich, Switzerland; 2 Department of Orthopedics, Balgrist University Hospital, University of Zurich, Zurich, Switzerland; 3 Institute for Biomechanics, ETH Zurich, Zurich, Switzerland

**Keywords:** Achilles tendon disease, Muscle, Fat fraction quantification, Magnetic resonance imaging, Two-point Dixon technique

## Abstract

**Objectives:**

To assess calf muscle constitution in chronic Achilles tendon disease (ATD) using two-point Dixon-based MRI (2pt-MRI_DIXON_).

**Materials and methods:**

This retrospective study analyzed 91 patients (36 females; 57.0 ± 14.4 years) with midportion or insertional chronic ATD who underwent clinical MRI of the Achilles tendon (AT), including 2pt-MRI_DIXON_ for quantitative assessment of calf muscle fat content (MFC). Additionally, two radiologists qualitatively assessed MFC, AT quality, and co-pathologies. 2pt-MRI_DIXON_-derived fat fractions (FF) were related to patients’ demographics and qualitative imaging findings.

**Results:**

The overall mean FF derived from 2pt-MRI_DIXON_ of the triceps surae muscle was 11.2 ± 9.3%. Comparing midportion and insertional ATD, there was no significant difference regarding fatty muscle infiltration assessed with 2pt-MRI_DIXON_ (*P* ≥ .47) or qualitative grading (*P* ≥ .059). More severe AT thickening (11 vs.9 mm, *P* < .001) and complete tears (29 vs. 9%, *P* = .025) were significantly more common in midportion ATD, while partial tears were significantly more frequent in insertional ATD (55 vs. 31%, *P* = .027). Soleus muscle edema was more prevalent in midportion than insertional ATD (40 vs. 9%, *P* = .002). In contrast, insertional ATD more commonly featured bone marrow edema (61 vs. 2%), Haglund’s deformity (67 vs. 0%), and retrocalcaneal bursitis (82 vs. 43%) (*P* ≤ .002). Significant correlations (*P* ≤ .001) were demonstrated between FF, AT diameter, age (both in midportion and insertional ATD), and body mass index (in midportion ATD only) (*ρ* range = 0.53–0.61).

**Conclusion:**

In chronic ATD, calf MFC was statistically equivalent (approximately 11%), irrespective of the localization of tendon damage. More severe tendon thickening and complete tears were more common in midportion ATD, and, vice versa, partial AT tears were significantly more frequent in insertional ATD.

**Supplementary Information:**

The online version contains supplementary material available at 10.1007/s00256-024-04845-7.

## Introduction

The Achilles tendon (AT) is the strongest tendon in the human body and is subjected to up to 12.5 times the body weight when running [1]. However, due to the high load on the AT and the constant strain during locomotion, it is also one of the tendons most frequently affected by overuse injuries [2]. Regarding pathophysiology, several mechanisms that reduce tissue perfusion and cause mechanical irritation lead to tendon degeneration, typically summarized as “Achilles tendinopathy” [3]. Acknowledging some controversies regarding the nomenclature in the literature [3, 4], we use the term “Achilles tendon disease” (ATD), under which we summarize all pathological changes of the AT.

ATD causes significant functional impairment and morbidity both in the general population and in athletes and constitutes a challenging condition often unresponsive to treatment protocols [2, 5, 6]. ATD can be categorized into two distinct localizations, namely midportion (MP) ATD, which is defined as a tendon pathology located 2–7 cm proximal to the calcaneal insertion, and insertional (IN) ATD, which means a tendon pathology less than 2 cm proximal to the calcaneus [7]. These manifestations are considered different in terms of underlying pathophysiology, clinical features, and response to treatment [8–12]. For example, patients with IN ATD usually have more severe symptoms and experience more significant disability than patients with MP ATD [9]. Furthermore, non-surgical therapy has been demonstrated to be less successful for IN ATD, a typical recalcitrant disease [10, 13–15]. However, irrespective of the ATD subtype, altered mechanical characteristics of the AT have significant implications for functional performance and further injury, as the AT has an essential influence on force transmission and power generation during the propulsive push-off phase of walking [16]. Likewise, previous research has demonstrated that ATD alters the mechanical and material properties of the AT, leading to fatty infiltration of the calf muscles, which probably results from altered musculoskeletal function [2, 17]. Vice versa, it is assumed that impaired muscle function is a risk factor for ATD [18], and, similarly to the rotator cuff in the shoulder, the quality of the triceps surae muscle might be a predictive factor for the outcome after AT treatment [19, 20].

Given this background, accurate quantification of calf muscle fatty infiltration in these patients could provide an additional biomarker when assessing Achilles tendon disease, as there is a known link between tendon damage and muscle quality, similar to rotator cuff pathologies in the shoulder. Therefore, the objectives of this retrospective study were to assess calf muscle constitution in ATD and to evaluate potential differences in MFC for the two distinct manifestations of ATD (MP vs. IN) using two-point Dixon-based MRI (2pt-MRI_DIXON_) in comparison to qualitative assessment of calf muscle and AT quality.

## Materials and methods

### Study design and participants

This retrospective single-center study was approved by the local institutional review board (Cantonal Ethics Committee Zurich) and conducted according to national ethical standards and in adherence to the principles of the Declaration of Helsinki and its subsequent amendments.

### Patients

**Fig. 1:**
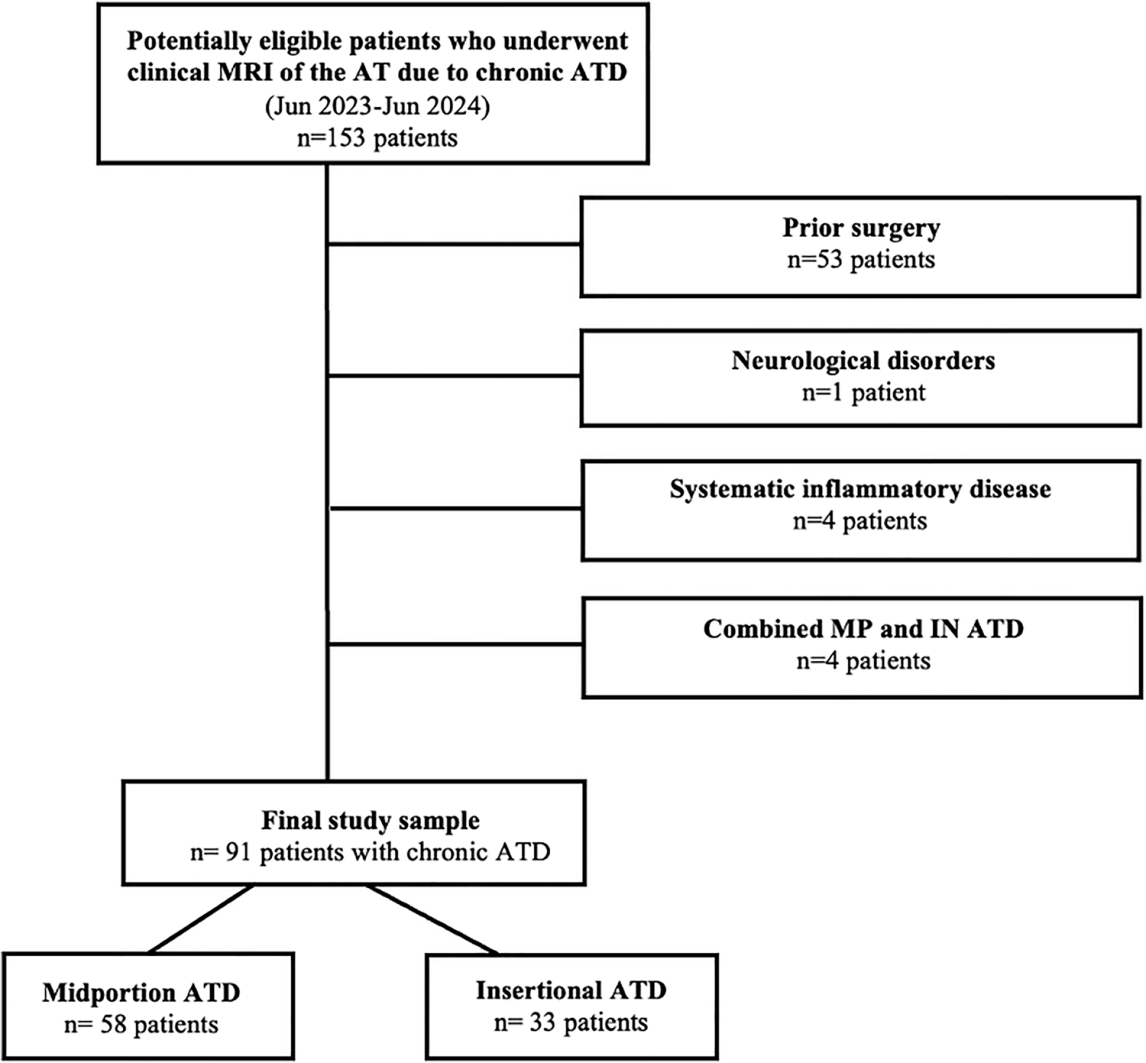
Flowchart illustrating the patient selection process and the study setup. Of 153 potentially eligible patients who underwent clinical MRI of the Achilles tendon (AT) due to chronic Achilles tendon disorders (ATD), 62 were excluded during the selection process. This resulted in a study sample of 91 patients with chronic ATD, of whom 58 had midportion ATD, and 33 had insertional ATD

All patients retrospectively included in the study had given written informed consent that allowed their health-related data to be used for research purposes. Patient charts were reviewed for patients who underwent clinical routine MRI of the AT and calf muscles due to chronic ATD between June 2023 and June 2024. Patients were eligible if they had a clinical diagnosis of chronic ATD with heel pain of at least 2 months by the referring physician and if MRI confirmed the clinical diagnosis (*n* = 91, Fig. 1). Imaging criteria for the diagnosis of ATD were applied according to current clinical standards [21, 22]. Prior surgery, neurological disorders, systematic inflammatory disease, and combined MP and IN ATD were exclusion criteria. Patients’ demographics and general clinical data were extracted from the patient charts.

### Imaging protocol

All patients underwent clinical routine MRI of the AT, including 2pt-MRI_DIXON_ at either 1.5-T (MAGNETOM Avanto Fit, Siemens Healthineers, *n* = 26 patients, MAGNETOM Sola, Siemens Healthineers, *n* = 19 patients) or 3.0-T (MAGNETOM Vida, Siemens Healthineers, *n* = 46 patients). Patients were examined in the supine position with a dedicated transmit-receive extremity coil for the AT and a phased-array body coil for imaging the calf muscles (both Siemens Healthineers). The imaging protocol consisted of our department’s clinical routine MRI protocol for examining patients with achillodynia, including an additional 2pt-MRI_DIXON_ sequence. The two-point Dixon gradient-echo (GRE) MR images were reconstructed with an automatic chemical-shift selective algorithm, which was shown to provide stable water- and fat-signal separation for dual-echo imaging using phase information to resolve the ambiguity of fat and water signal in chemical-shift imaging [23, 24]. Detailed scan parameters are given in Table 1.

**Table 1:** MRI protocol and corresponding sequence parameters

	Achilles tendon					Calf muscles		
Sequence	PDw fs TSE	PDw TSE	PDw fs TSE	T2w TSE	T2w fs TSE	T1w TSE	PDw fs TSE	2pt-Dixon GRE
Orientation	Sag	Cor	Cor	Tra	Tra	Tra	Tra	Tra
Number of dimensions	Two	Two	Two	Two	Two	Two	Two	Three
Repetition time (ms)	5000	4500	5000	3000	5060	500	4000	4.0
Echo time (ms)	32	33	33	91	84	10	39	1.3; 2.6
Flip angle (°)	125	125	125	135	135	135	135	3
Number of averages	1	1	1	1	1	1	1	2
Slice thickness (mm)	3	3	3	5	4	7.5	5	5
Matrix size	448 × 358	448 × 358	448 × 358	416 × 333	384 × 307	448 × 358	400 × 280	160 × 128
Bandwidth (Hz/px)	302	302	302	250	250	266	298	475
Number of images	28	36	36	26	26	52	78	72

*Cor*, coronal; *fs*, fat-saturated; *GRE*, gradient-echo; *Hz*, Hertz; *PDw*, proton-density-weighted; *px*, pixel; *sag*, sagittal; *tra*, transversal; *TSE*, turbo spin-echo; *T1w*, T1-weighted; *T2w*, T2-weighted; *2pt-Dixon*, two-point Dixon-based reconstruction of fat- and water-only images

The table shows the sequence parameters for the 3.0 T scanner. The sequence parameters for the two other scanners vary slightly

### Image analysis

#### Quantitative analysis

**Fig. 2:**
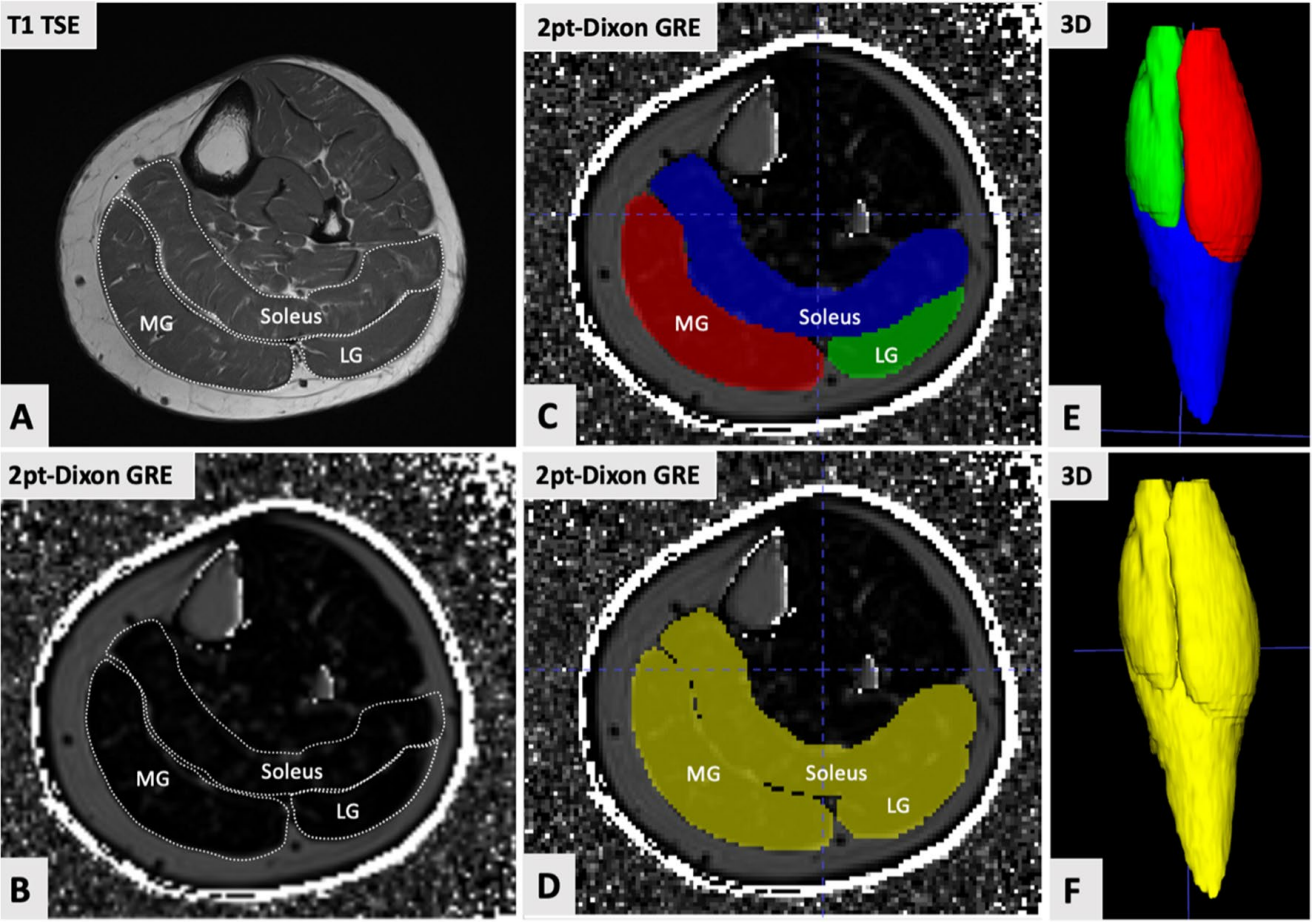
Quantitative volumetric assessment of calf muscle fat content (MFC) using the example of a 68-year-old female (left side). Outlining of calf muscles was done in conjunction with the corresponding transversal T1-weighted sequence (**A**). The reconstructed two-point Dixon gradient-echo (GRE) MR images were uploaded into ITK-Snap (**B**). Next, the medial gastrocnemius (MG), lateral gastrocnemius (LG), and soleus muscles were manually outlined on all slices (**C**). After that, automated three-dimensional (3D) reconstructions were generated with an output of the mean volumetric fat fraction (FF) and whole muscle volume (WMV) for each muscle separately. In this case, the MG muscle demonstrated a FF of 10.3% (WMV = 145.8 cm^3^), the LG muscle of 8.6% (WMV of 85.3 cm^3^), and the soleus muscle of 10.7% (WMV = 211.2 cm^3^). These segmentations (MG, LG, and soleus muscles) were summed up to determine the FF and WMV of the triceps surae muscle (**D**). In this example, the FF of the triceps surae muscle was calculated with 10.2% (WMV = 442.3cm^3^). **E**, **F** The corresponding 3D reconstruction images

Quantitative image analysis was done using ITK-Snap (v. 4.2.0) [25] in conjunction with a Picture Archiving and Communication System (PACS) workstation (Merlin, Phoenix-PACS). The reconstructed two-point Dixon GRE MR images were anonymized and exported from the PACS as Neuroimaging Informatics Technology Initiative (NIfTI) files and uploaded into the ITK-Snap software, which was used for determining quantitative FF values of the triceps surae muscle. This was done by one musculoskeletal fellowship-trained radiologist with five years of experience (SSG) as follows: In the first step, the medial gastrocnemius (MG), lateral gastrocnemius (LG), and soleus muscles were manually outlined on all slices of the transversal reconstructed two-point Dixon GRE MR images. Each muscle was outlined with the corresponding transversal T1-weighted sequence of the calf muscles for better anatomical correlation. Tendon insertions were excluded from the volumetric assessment. After this, automated three-dimensional (3D) reconstructions were generated with an output of the mean volumetric FF and whole muscle volumes (WMV) for each muscle separately. In the next step, the MG, LG, and soleus muscle segmentations were summarized and evaluated to determine the 3D FF and WMV of the triceps surae muscle (Fig. 2).

#### Qualitative analysis

Two independent musculoskeletal fellowship-trained radiologists (SSG and GWK, with 5 and 6 years of experience) conducted qualitative image analysis. Both readers were blinded to clinical data and each other’s results. All images were interpreted on a PACS workstation (Merlin, Phoenix-PACS) in random order and were presented to the radiologists with the minimum of annotations allowed by the software.

First, readers assessed the quality of the AT on a three-point scale based on Schweitzer et al. [4] with the following specifications adapted to our cohort: (1) “tendinosis,” tendon thickening, convex spindle-shape, normal signal or hyperintense areas on T1-weighted images; (2) “partial tear,” tendon thickening, hyperintense on T1- and T2-weighted images; (3) “complete tear,” discontinuity of the AT. In addition, AT thickening was assessed by measuring the tendon’s maximum anterior to posterior diameter on sagittal fat-saturated (fs) proton-density (PD)-weighted images.

Next, readers visually determined the extent of fatty infiltration of the MG, LG, and soleus muscles on T1-weighted images using the Goutallier classification (grades 0–4) [26].

Lastly, readers assessed co-pathologies, including MG, LG, and soleus muscle edema, Kager’s fat pad edema, Haglund’s deformity, bone marrow edema in the posterosuperior calcaneus, and retrocalcaneal bursitis following established criteria of MR image interpretation [21, 22].

### Statistical analysis

Statistical analyses were performed in SPSS Statistics (v. 29, IBM Corporation). Quantile–Quantile plots and the Shapiro–Wilk test were used to test for normal distribution of continuous variables. In addition to descriptive statistics, the Chi-square and Mann–Whitney *U* tests were used to evaluate differences between patients with MP and those with IN ATD. The Spearman’s rank correlation coefficient (*ρ*) was used to measure the strength and direction of correlation between nonparametric variables. The inter-reader agreement was analyzed using intraclass correlation coefficients (ICC) and kappa statistics (Cohen’s *κ*). The level of agreement was reported as follows [27]: 0.0 = poor, 0.01–0.20 = slight, 0.21–0.40 = fair, 0.41–0.60 = moderate, 0.61–0.80 = substantial, > 0.80 = almost perfect. All statistical tests were performed two-sided, and a level of significance (*α*) below 0.05 was used.

## Results

### Demographics

The study included 91 patients (36 females, mean age 57.0 ± 14.4 years). Table 2 provides detailed general demographic data of the overall study cohort and patients with MP compared to IN ATD.

**Table 2:** Patient demographics and general clinical data of the overall study cohort and patients with midportion (MP) compared to insertional (IN) Achilles tendon disease (ATD)

	Overall study cohort	MP ATD	IN ATD	*P*-value^1^
Number of patients	91 (100)	58 (63.7)	33 (36.3)	
Female sex	36 (39.6)	22 (37.9)	14 (42.4)	.67
Right side	43 (47.3)	31 (53.5)	12 (36.4)	.12
Age (years)	57.0 ± 14.4	57.2 ± 14.5	56.7 ± 14.5	.78
Height (m)	1.74 ± 0.08	1.74 ± 0.08	1.74 ± 0.08	.88
Body mass (kg)	83.9 ± 17.6	81.8 ± 17.3	87.8 ± 17.8	.12
Body mass index (kg/m^2^)	27.7 ± 5.3	26.9 ± 4.9	29.1 ± 5.7	.095

The number of patients is given as a frequency with percentages in parentheses. Continuous data are presented as mean ± standard deviation

^1^*P*-value for comparison of demographics and general clinical data in patients with MP and IN ATD by chi-square and Mann–Whitney *U* tests, respectively

*ATD*, Achilles tendon disease; *IN*, insertional; *MP*, midportion

### Quantitative analysis of calf muscle fat content

Volumetric 3D FF derived from 2pt-MRI_DIXON_ and corresponding WMV were obtained in 91 patients within the MG, LG, and soleus muscles. The mean FF in the overall study cohort within the triceps surae muscle was 11.2 ± 9.3% with a mean WMV of 761.5 ± 196.2 cm^3^. In the overall study cohort, the mean FF of the MG muscle was 10.7 ± 11.1% (mean WMV 194.6 ± 59.1 cm^3^), of the LG muscle, it was 8.6 ± 6.6.% (mean WMV 125.0 ± 36.2 cm^3^), and of the soleus muscle, it was 12.1 ± 10.3% (mean WMV 441.8 ± 129.4 cm^3^).

Mean volumetric FF and WMV in MP compared to IN ATD for the triceps surae muscle and separate muscles (MG, LG, soleus muscle) as derived from 2pt-MRI_DIXON_ in comparison with mean values of visual qualitative grading according to Goutallier are given in Table 3. There were no significant differences when comparing the MP and IN cohorts’ quantitative and qualitative mean values (*P* ≥ 0.22).

**Table 3:** Mean volumetric fat fractions (FF) and whole muscle volumes (WMV) in midportion (MP) compared to insertional (IN) Achilles tendon disease (ATD) for separate muscles as derived from two-point Dixon-based MRI (2pt-MRI_DIXON_) in comparison with mean values of visual qualitative grading according to Goutallier

	MP ATD	IN ATD	*P*-value
Triceps surae			
FF_DIXON_ (%)	10.9 ± 8.7	11.7 ± 10.3	.74
WMV (cm^3^)	759.0 ± 186.4	765.9 ± 215.2	.69
Medial gastrocnemius			
FF_DIXON_	11.1 ± 12.2	10.1 ± 8.9	.47
WMV	188.4 ± 50.3	205.5 ± 71.6	.22
Goutallier grading (0–4)*	1.4 ± 0.7	1.4 ± 0.9	.92
Lateral gastrocnemius			
FF_DIXON_	8.3 ± 7.1	8.9 ± 5.7	.50
WMV	126.1 ± 34.7	123.2 ± 39.0	.69
Goutallier grading (0–4)*	1.3 ± 0.6	1.4 ± 0.8	.46
Soleus			
FF_DIXON_	11.6 ± 9.0	13.2 ± 12.4	.88
WMV	444.5 ± 130.2	437.2 ± 129.6	.88
Goutallier grading (0–4)*	1.5 ± 0.7	1.6 ± 0.9	.88

Continuous data are presented as mean ± standard deviation

^*^Visual grades ranged from 0 to 4, according to the Goutallier classification [26]

*ATD*, Achilles tendon disease; *FF*_*DIXON*_, volumetric 3D fat fractions derived from 2pt-MRI_DIXON_; *IN*, insertional; *MP*, midportion; *WMV*, whole muscle volume

### Qualitative analysis

#### Visual assessment of calf muscle fat content

**Fig. 3:**
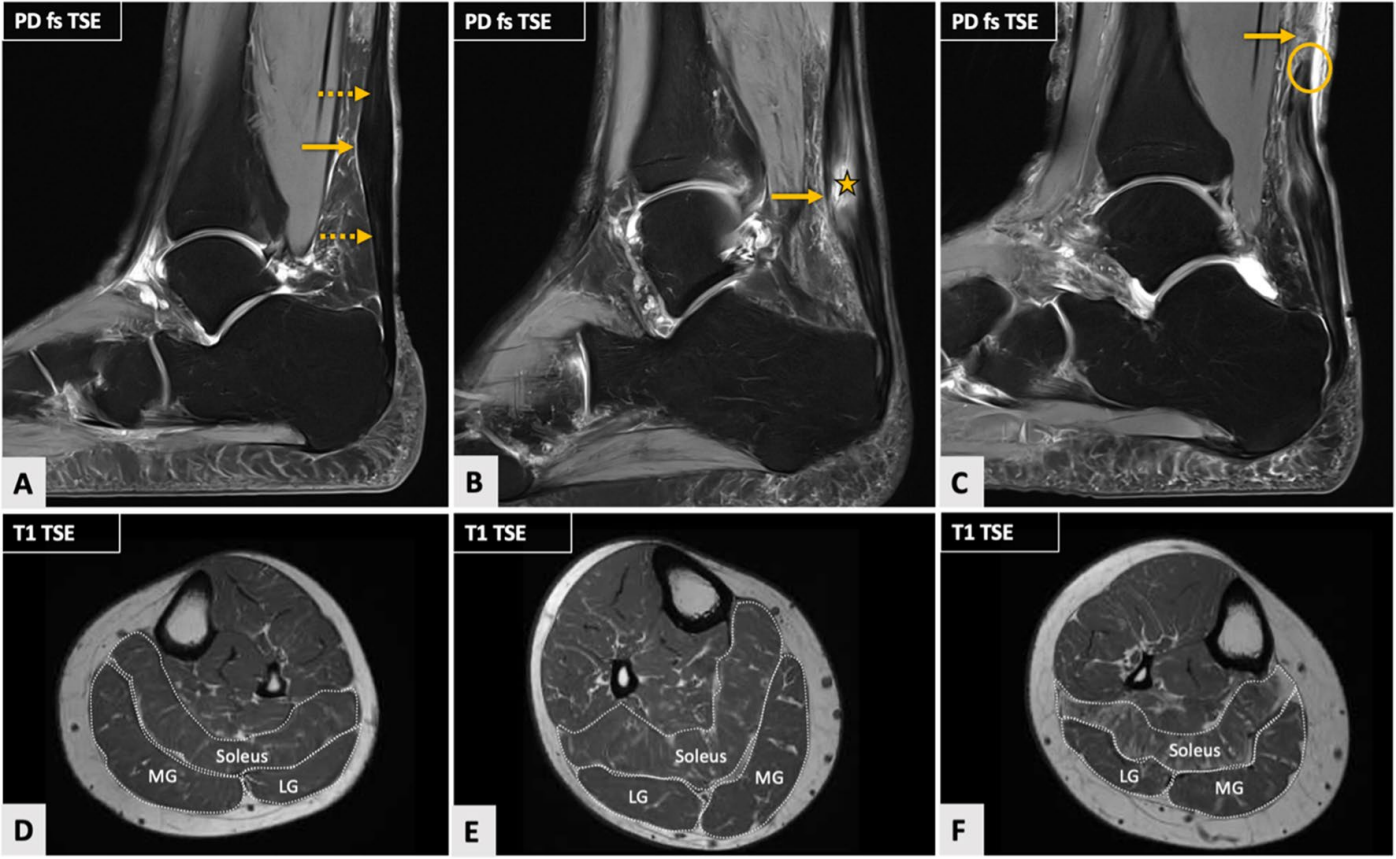
Three cases of midportion (MP) Achilles tendon disease (ATD) with increasing severity of structural Achilles tendon (AT) damage as demonstrated on sagittal fat-saturated (fs) proton-density (PD)-weighted images (**A–C**) and corresponding transversal T1-weighted images of calf muscles at the level of the middle third of the lower leg (**D–F**) representatively illustrating fatty muscle infiltration visually assessed by using the Goutallier classification in comparison to quantitatively determined muscle fat content (MFC). Image **A** shows a case of left-sided MP ATP with spindle-shaped thickening of the AT at the level of the middle third of the tendon (arrow) in a 68-year-old female. Minimal hyperintense signal alterations within the tendon substance can be seen at the proximal and distal ends of the spindle-shaped thickening (dashed arrows). In **B** (right AT of a 58-year-old male), there is even more severe spindle-shaped thickening of the AT (arrow) and a partial tear of the AT at the middle third of the tendon. In contrast, a complete AT rupture in a 62-year-old male (right side) is visible in **C**, where one can depict a discontinuity of the AT at the level of the middle tendon third (arrow). The distal tendon stump has comparatively smooth edges (circle). In the first case (**D**), visually assessed fatty infiltration of the medial (MG) and lateral gastrocnemius (LG) muscles was rated as a grade 1, whereas that of the soleus muscle was rated as a grade 2. Fatty muscle infiltration of the LG muscle in the second case (**E**) was assessed as a grade 1, while the MG and soleus muscle fatty infiltration were rated as grade 2. In the third case (**F**), the degree of fatty infiltration of the MG and LG muscles was evaluated as a grade 2, while the soleus muscle was assessed as a grade 3. Quantitative fat fraction values were 10.3%, 8.6%, and 10.7% (MG, LG, soleus muscle) in the first case (**A**, **D**); 11.1%, 9.4%, and 12.2% in the second case (**B**, **E**); and 14.1%, 9.3%, and 13.9% in the third case (**C**, **F**). These case examples underline the inferior discriminatory power of qualitative compared to quantitative MFC determination

**Fig. 4:**
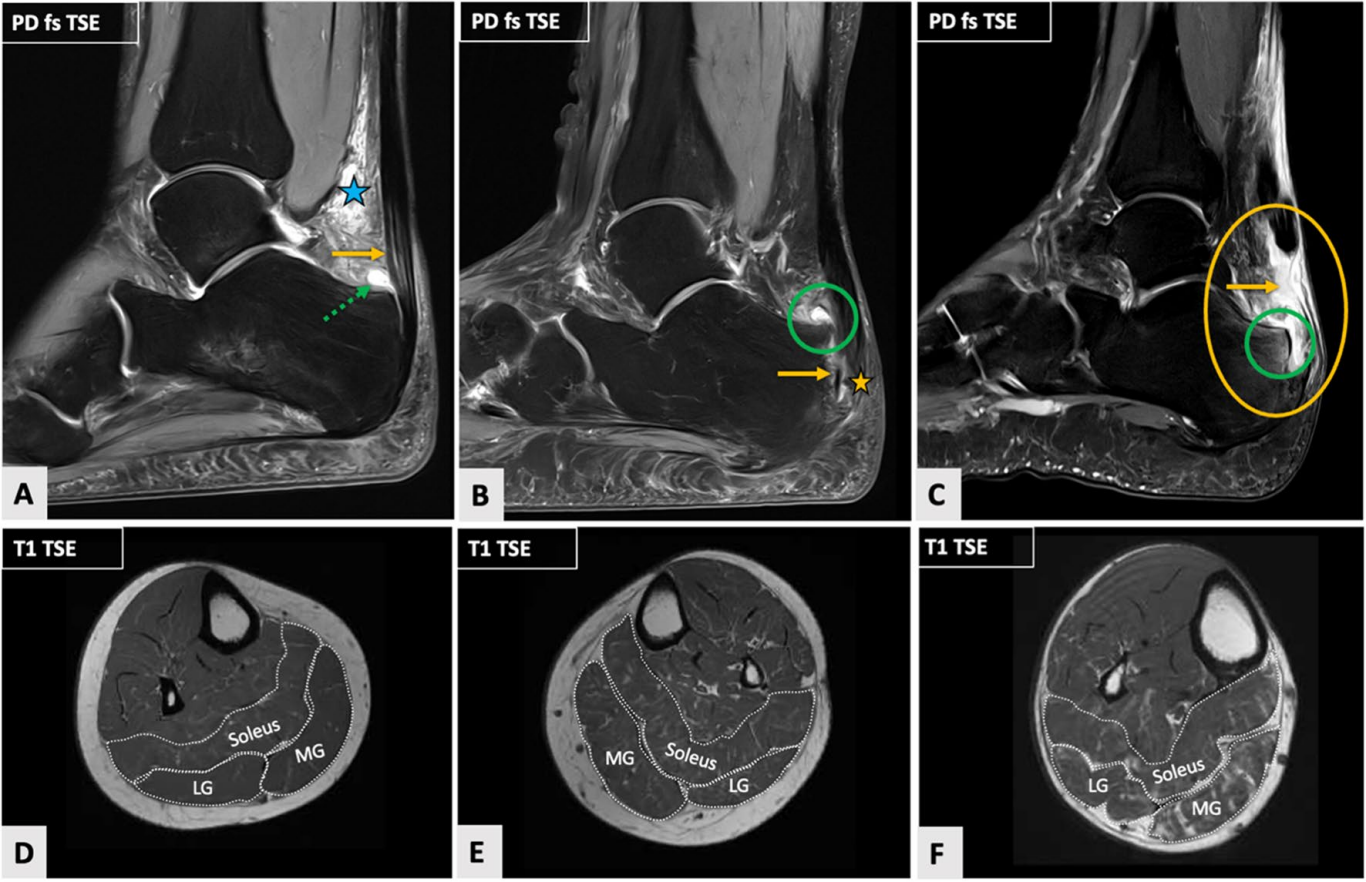
Three cases of insertional (IN) Achilles tendon disease (ATD) with increasing severity of structural Achilles tendon (AT) damage as demonstrated on sagittal fat-saturated (fs) proton-density (PD)-weighted images (**A**–**C**) and corresponding transversal T1-weighted images of calf muscles at the level of the middle third of the lower leg (**D**–**F**) representatively illustrating fatty muscle infiltration visually assessed by using the Goutallier classification [26] in comparison to quantitatively determined muscle fat content (MFC). Image **A** depicts IN ATD in a 45-year-old female (right side), which is characterized by tendon thickening and minimal intratendinous hyperintensities on fluid-sensitive images (arrow). There is severe inflammation of the paratendinous soft tissues with significant Kager’s fat pad edema (asterisk) and retrocalcaneal bursitis (dashed arrow). Image **B** illustrates IN ATD in a 54-year-old female (left side) with a partial rupture at the tendon insertion (arrow, asterisk), a Haglund’s deformity of the calcaneus and retrocalcaneal bursitis (circle), but no Kager’s fat pad edema. A case of IN ATD with complete rupture of the AT in a 72-year-old male (right side) is shown in image (**C**). There is discontinuity of the AT directly at its insertion (arrow, yellow circle). In addition, there is mild Haglund’s deformity with edema in the posterosuperior part of the calcaneus (green circle) and extensive fluid in the soft tissues. In the first patient example (**D**), visually assessed fatty infiltration of the medial (MG) and lateral gastrocnemius (LG) muscles was rated as a grade 1, whereas that of the soleus muscle was rated as a grade 2. In the second case (**E**), visual assessment of fatty muscle infiltration was assessed as a grade 1 for all three muscles. In the third case (**F**), the degree of fatty infiltration of the LG and soleus muscles was evaluated as a grade 2, while the MG muscle was assessed as a grade 3. Quantitative fat fraction values were 4,7%, 4.6%, and 6.9% (MG, LG, soleus muscle) in the first case (**A**, **D**); 9.3%, 9.2%, and 9.5% in the second case (**B**, **E**); and 21.1%, 11.0%, and 13.6% in the third case (**C**, **F**). Analogous to Fig. 3, these case examples underline the superiority of quantitative over qualitative MFC determination

For the two ATD types, visual assessment of calf MFC is illustrated in Figs. 3 and 4. Table 4 displays detailed results of the qualitative visual analysis of MFC for patients with MP compared to IN ATD. There were no significant differences in the degree of fatty infiltration of the MG, LG, and soleus muscles between the two groups (*P* ≥ 0.059).

**Table 4:** Results of qualitative visual assessment of fatty degeneration of the gastrocnemii and soleus muscles in patients with midportion (MP) compared to insertional (IN) Achilles tendon disease (ATD)

	MP ATD	IN ATD
Medial gastrocnemius		
Grade 0	4 (6.9)	4 (12.1)
Grade 1	29 (50.0)	15 (45.5)
Grade 2	23 (39.7)	11 (33.3)
Grade 3	1 (1.7)	2 (6.1)
Grade 4	1 (1.7)	1 (3.0)
Lateral gastrocnemius		
Grade 0	4 (6.9)	3 (9.1)
Grade 1	33 (56.9)	15 (45.5)
Grade 2	21 (36.2)	14 (42.4)
Grade 3	0 (0)	0 (0)
Grade 4	0 (0)	1 (3.0)
Soleus		
Grade 0	2 (3.5)	2 (6.1)
Grade 1	28 (48.3)	15 (45.5)
Grade 2	24 (41.4)	12 (36.4)
Grade 3	4 (6.9)	2 (6.1)
Grade 4	0 (0)	2 (6.1)

The number of patients is given as a frequency with percentages in parentheses

The extent of fatty muscle infiltration was assessed on T1-weighted images using the 5-point scale according to Goutallier [26]: grade 0, completely normal muscle without any fatty streaks; grade 1, muscle containing some fatty streaks; grade 2, muscle tissue > fat tissue; grade 3, muscle tissue equals fat tissue; grade 4, fatty tissue > muscle tissue

*ATD*, Achilles tendon disease; *IN*, insertional; *MP*, midportion

In addition, Table 5 provides detailed results for mean 2pt-MRI_DIXON_-derived FF with corresponding ranges of the gastrocnemii and soleus muscles for each Goutallier grades 0–4 for patients with MP compared to IN ATD. With increasing Goutallier grades, the MP and the IN ATD groups show continuously increasing FF values. Still, FF values were found to have a wide range and overlap between the distinct Goutallier grades.

**Table 5:** Mean FF derived from two-point Dixon-based MRI (2pt-MRI_DIXON_). Data are presented with the corresponding minimum to maximum FF values of the gastrocnemii and soleus muscles between Goutallier grades 0–4 derived from visual grading of muscle fat content (MFC) for patients with midportion (MP) compared to insertional (IN) Achilles tendon disease (ATD)

	*n*_MP_	FF_MP_	*n*_IN_	FF_IN_
Medial gastrocnemius				
Grade 0	4	4.9 (3.5–6.8)	4	3.2 (2.6–3.7)
Grade 1	29	6.3 (2.9–11.2)	15	6.3 (4.1–12.5)
Grade 2	23	13.0 (5.8–22.1)	11	12.4 (5.6–21.1)
Grade 3	1	72.2 (72.2–72.2)	2	19.1 (17.1–21.1)
Grade 4	1	69.3 (69.3–69.3)	1	49.2 (49.2–49.2)
Lateral gastrocnemius				
Grade 0	4	4.4 (3.4–5.3)	3	2.9 (2.4–3.3)
Grade 1	33	5.9 (3.1–11.6)	15	6.5 (4.3–12.0)
Grade 2	21	12.8 (6.2–55.4)	14	11.2 (6.6–18.7)
Grade 3	0	NA	0	NA
Grade 4	0	NA	1	32.6 (32.6–32.6)
Soleus				
Grade 0	2	3.9 (3.6–4.1)	2	3.2 (2.9–3.5)
Grade 1	28	6.0 (3.6–8.6)	15	7.0 (3.6–11.5)
Grade 2	24	15.0 (7.6–30.5)	12	14.0 (8.8–23.1)
Grade 3	4	33.6 (19.6–51.5)	2	23.9 (13.6–34.2)
Grade 4	0	NA	2	54.0 (49.4–58.5)

The first column displays Goutallier grades 0–4 [26] for each muscle assessed

*n* gives the number of patients with midportion (*n*_MP_) and insertional Achilles tendon disease (*n*_IN_)

Continuous data (FF) are presented as mean with range in parentheses

*ATD*, Achilles tendon disease; *IN*, insertional; *NA*, not applicable; *MP*, midportion

#### Achilles tendon quality

Tendinosis and structural damage of the AT (partial and complete AT tears) in MP and IN ATD are illustrated in Figs. 3 and 4. Tendinosis was present in a total of 35/91 patients (38.5%), whereas a partial tear was observed in 36/91 patients (39.6%), and a complete tear was seen in 20/91 patients (21.9%). Subgroup analysis of patients with MP vs. IN ATD demonstrated a similar distribution of tendinosis (MP, 23 (39.7%) vs. IN, 12 (36.4%) patients; *P* = 0.76), while a partial tear of the AT was significantly more often observed in IN ATD (MP, 18 (31.0%) vs. IN, 18 (54.6%) patients; *P* = 0.027), and, vice versa, a complete tear of the AT was significantly more frequently seen in MP ATD (MP, 17 (29.3%) vs. IN, 3 (9.1%) patients; *P* = 0.025). Additionally, patients with MP ATD had a significantly thicker AT compared to those with IN ATD (MP, 11.2 ± 2.8 mm (range 6.4–19.3 mm) vs. IN, 8.9 ± 2.5 mm (range 5.4–14.9 mm); *P* < 0.001). No significant differences in FF values of the triceps surae muscle were seen between patients with tendinosis (grade 1) compared to those with structural damage of the AT (grades 2 and 3) (*P* = 0.26).

#### Assessment of co-pathologies

**Fig. 5:**
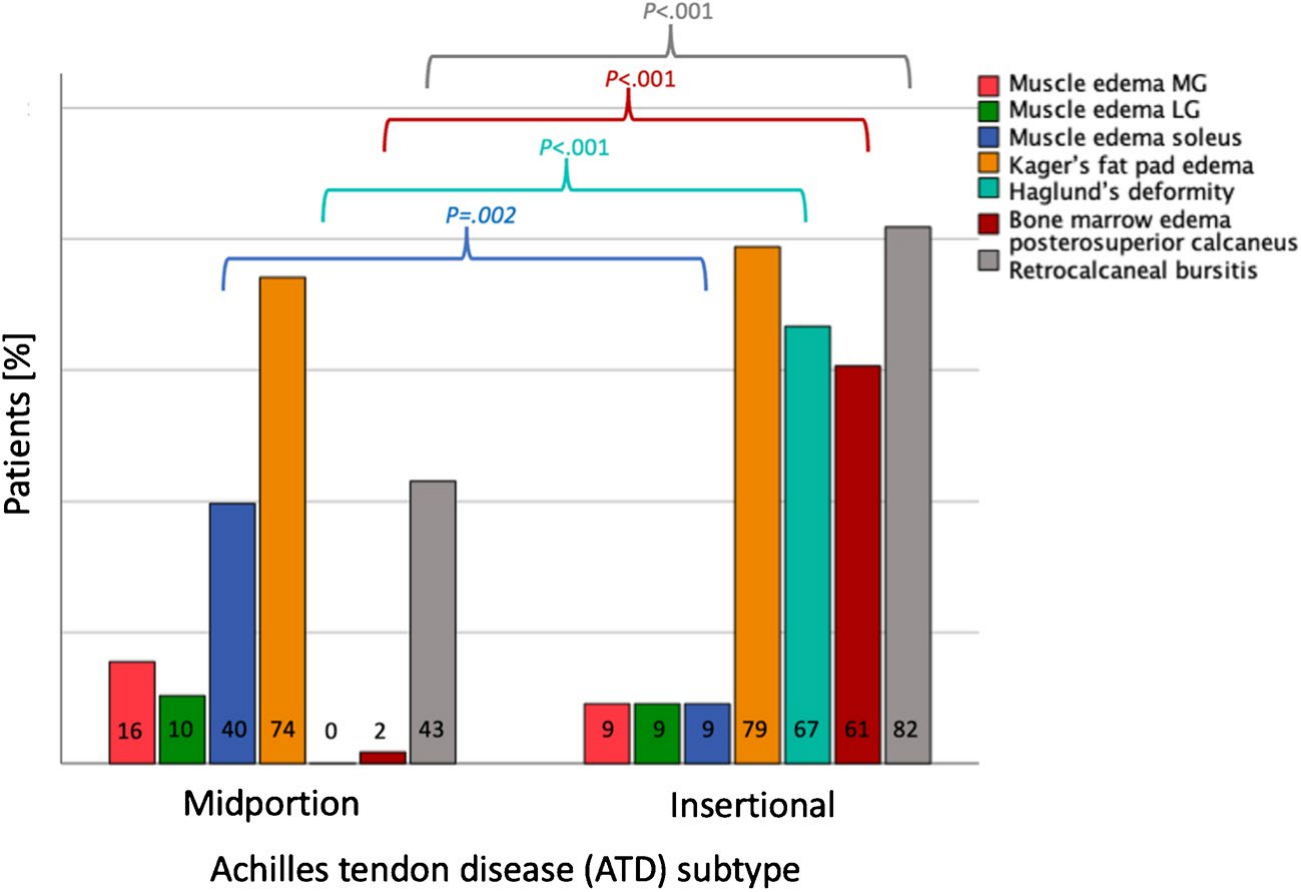
Assessment of co-pathologies in 58 patients with midportion (MP) vs. 33 patients with insertional (IN) Achilles tendon disease (ATD). Soleus muscle edema was significantly more common in patients with MP than IN ATD (*P* = .002). Haglund’s deformity, bone marrow edema in the posterosuperior calcaneus, and retrocalcaneal bursitis (all *P* < .001) were significantly more often observed in patients with IN than MP ATD. No significant differences between the two subtypes of ATD were found for edema in the MG or LG muscle and Kager’s fat pad edema

Soleus muscle edema was diagnosed in 26/91 patients (28.6%) and was significantly more common in patients with MP compared to IN ATD (MP, 23 (39.7%) vs. IN, 3 (9.1%) patients; *P* = 0.002), while MG (MP, 9 (15.5%) vs. 3 (9.1%) patients; *P* = 0.39) and LG muscle edema occurred comparably often in both groups (MP, 6 (10.3%) vs. 3 (9.1%) patients, *P* = 0.85). The presence of Kager’s fat pad edema was similar in patients with MP and those with IN ATD (MP, 43 (74.1%) vs. 26 (78.8%) patients; *P* = 0.62). Haglund’s deformity (MP, 0 vs. 22 (66.7%) patients; *P* < 0.001), bone marrow edema in the posterosuperior calcaneus (MP, 1 (1.7%) vs. 20 (60.6%) patients; *P* < 0.001), and retrocalcaneal bursitis (MP, 25 (43.1%) vs. 27 (81.8%) patients; *P* < 0.001) were significantly more often observed in patients with IN compared to MP ATD. The frequencies of assessed co-pathologies for MP and IN ATD are displayed in Fig. 5.

#### Inter-reader agreement

The inter-reader agreement was almost perfect for measuring the diameter of the AT in the sagittal plane (ICC 0.93), whereas it was substantial to almost perfect for assessing AT quality, fatty degeneration of calf muscles by visual grading, and co-pathologies (*κ* range 0.75–0.93). Detailed results are provided in Supplementary Table 1.

### Correlation between FF, patients’ demographics, qualitative parameters, and co-pathologies

The Spearman’s rank correlation coefficient (*ρ*) revealed a significant correlation between FF of the triceps surae muscle for AT diameter in MP (*P* = 0.005) and IN (*P* = 0.001) ATD; however, the correlation strengths were weak (*ρ* = 0.37) and moderate (*ρ* = 0.54), respectively. Also, there was a correlation between FF of the triceps surae muscle and patients’ age in MP (*P* < 0.001) and IN (*P* < 0.001) ATD with moderate (*ρ* = 0.56) and strong correlation coefficients as well as patients’ BMI in MP ATD (*P* < 0.001) with moderate strength (*ρ* = 0.53). AT quality, patients’ gender, and BMI in IN ATD, in contrast, did not show significant correlations with FF of the triceps surae muscle (*P* ≥ 0.15).

## Discussion

This study used two-point Dixon-based MRI (2pt-MRI_DIXON_) to assess calf muscle constitution in Achilles tendon disease (ATD) and demonstrated a mean FF of 11% within the triceps surae muscle, irrespective of the disease localization (midportion vs. insertional). Looking at individual calf muscles, the highest mean FF was found within the soleus muscle (12%), followed by the MG (11%) and LG (9%) muscles. Nevertheless, the two subtypes differed: the MP ATD, compared to the IN ATD, involved more severe tendon thickening and complete tears, whereas partial tears occurred more often at the tendon insertion. Furthermore, looking at co-pathologies, soleus muscle edema was more prevalent in MP ATD. In contrast, Haglund’s deformity, bone marrow edema in the posterosuperior calcaneus, and retrocalcaneal bursitis were more common in insertional tendon disease.

Previous studies have suggested that MFC assessment may be a biomarker for detecting muscular insufficiency and predicting postoperative outcomes in various degenerative musculoskeletal diseases [17, 19, 20, 26, 28–32]. Thus, 2pt-MRI_DIXON_ has been applied for assessing MFC of the lumbar muscles in patients with lower back pain [29], for assessing fatty degeneration of the gastrocnemius and tibialis anterior muscles in patients with achillodynia [30], or, recently, for determining fatty degeneration of the rotator cuff muscles as a basis for assessing early supraspinatus muscle atrophy [28]. With this, 2pt-MRI_DIXON_ has been shown to be an accurate and reliable method for quantifying MFC, allowing fast and volumetric data acquisition [29]. However, the results of 2pt-MRI_DIXON_ may be affected by some measurement biases, such as T2* and T1 bias, which may impair the accuracy of MFC determination [33]. Yet, previous studies on muscle/fat phantoms or MR spectroscopy as the standard of reference proved that 2pt-MRI_DIXON_ allows for accurate quantification of MFC [30, 33–36]. For example, Fischer et al. determined FF within the gastrocnemius and tibialis anterior muscles and were able to show that 2pt-MRI_DIXON_-derived FF correlated linearly and highly with the actual fat content in a dedicated phantom in a range from 0 to 100% MFC [30]. Furthermore, in the same study, the authors showed that 2pt-MRI_DIXON_-derived FF closely and significantly matched FF derived from MR spectroscopy [30].

In patients with ATD, we demonstrated a mean FF of 11% within the MG muscle, 9% within the LG muscle, and 12% within the soleus muscle. These values are higher than the analysis by Fischer et al., who analyzed FF of the calf muscles in 30 patients with achillodynia (mean age 57 years) compared to 20 healthy volunteers (mean age 30 years) and reported a mean FF of about 7% of the gastrocnemius muscle in patients and a significant lower FF of about 4% in healthy volunteers [30]. Another study evaluated age-related differences in muscle volume, intramuscular fat, and mechanical properties in the triceps surae muscle and was able to show that older adults (mean age 70 years) displayed significantly higher levels of intramuscular fat, yet similar volumes of the MG, LG, and soleus muscles, compared to younger adults (mean age 25 years) [32]. With this, the authors reported FF of about 7% for the MG, LG, and soleus muscles in young, healthy adults, whereas FF of 12%, 10%, and 12% were found for these muscles in older but also healthy individuals [32]. Interestingly, the FF values that Pinel et al. determined for older, healthy individuals are similar to those we determined for patients with ATD. In their study [32], the authors also used ITK-Snap for manual 3D segmentation of calf muscles and 2pt-MRI_DIXON_ for FF determination; however, their sequence parameters of the 2pt-MRI_DIXON_ were not exactly the same compared to ours, e.g., they used a flip angle of 10° [32], which leads to a higher risk of T1 bias with 2pt-MRI_DIXON_ compared to our lower flip angle of 3°. In contrast, Fischer et al. calculated FF by placing a single region of interest in the gastrocnemius and tibialis anterior muscles on both the fat- and water-only images corresponding to spectroscopic voxels [30], which limits comparability with our FF results, as we segmented the muscles as a whole to determine FF.

Regarding the technique of MFC assessment, there is a consensus between previous studies and ours, as all suggest that quantitative methods are superior to qualitative evaluation of MFC [30, 37]. Although we determined continuously increasing mean FF values with increasing Goutallier grades, there were large ranges and a vast overlap of FF values for each distinct Goutallier grade (see Table 5), underlining that MFC assessment is more accurate using quantitative techniques. As visual assessment of fatty muscle infiltration using the Goutallier classification is based on an ordinal scale with only five subtypes, it must be assumed that there is an inherent lack of discriminatory power; furthermore, in contrast to quantitative MFC evaluation, visual assessment has been reported to hamper the detection of subtle fatty muscle infiltration [30].

In contrast to Fischer et al., who reported a significantly higher MFC in individuals with structural damage of the AT compared to those with intact tendons [30], there were no significant differences in FF values of the triceps surae muscle between patients with tendinosis (grade 1) compared to those with structural damage of the AT (grades 2 and 3) in our study. However, it is essential to note that, in their study, there were also individuals with normal AT included (5/30, 17%). In contrast, we only included patients with ATD, confirmed by clinical examination and MRI. Moreover, the number of patients with tendinosis (33 vs. 39%), partial (23 vs. 40%), and complete AT rupture was also different between their study and ours (13 vs. 22%).

Our study showed that the two subtypes of ATD (MP vs. IN) go along with different co-pathologies typical for the respective manifestation. Soleus muscle edema was diagnosed in almost a third of cases (28.6%) and was significantly more common in patients with MP than IN ATD (40 vs. 9%). In contrast, Haglund’s deformity (0 vs. 67%), bone marrow edema in the posterosuperior calcaneus (2 vs. 61%), and retrocalcaneal bursitis (43 vs. 82%) were significantly more often observed in patients with IN compared to MP ATD. These co-pathologies should be considered when analyzing clinical AT MR examinations.

We demonstrated a positive correlation between the FF of the triceps surae muscle, patients’ age in MP and IN ATD, and patients’ BMI in MP ATD. Furthermore, there was a significant correlation between the FF of the triceps surae muscle for AT diameter in MP and IN ATD; however, the correlation strengths were weak and moderate, respectively.

Some limitations of our study need to be addressed. First, we used a PD-weighted two-point Dixon sequence instead of a multi-point Dixon MR sequence [38]. However, we did not see those potential errors associated with 2pt-MRI_DIXON_ to significantly impair FF values, which aligns with previous studies evaluating both two-point and multi-point Dixon MRI for intramuscular fat quantification [29, 30]. Second, patients were examined using different scanners and field strengths, which might add a potential bias. Third, due to the retrospective study design, we only assessed MFC in patients with ATD who had a clinical indication for MRI and did not examine healthy individuals without clinical symptoms and structural tendon damage.

In summary, this study demonstrates a calf muscle fat content assessed with 2pt-MRI_DIXON_ of about 11% in chronic Achilles tendon disease, irrespective of the subtype (midportion vs. insertional). These results can serve as an additional biomarker when assessing Achilles tendon disease, as there is a known link between tendon damage and muscle quality, similar to rotator cuff pathologies in the shoulder.

## Supplementary Information

Below is the link to the electronic supplementary material.

Supplementary file1 (DOCX 17 KB)

## Data Availability

This study’s data is available by the corresponding author upon reasonable request.

## Abbreviations

AT: Achilles tendon
ATD: Achilles tendon disease
BMI: Body mass index (= body mass [kg]/height [m^2^])
FF: Fat fraction
GRE: Gradient-echo
IN: Insertional
LG: Lateral gastrocnemius (muscle)
MFC: Muscle fat content
MG: Medial gastrocnemius (muscle)
MP: Midportion
PACS: Picture Archiving and Communication System
WMV: Whole muscle volume
2pt-MRI_DIXON_: Two-point Dixon-based MRI
3D: Three-dimensional
