# Supplementary material for: Assessment of calf muscle constitution in chronic Achilles tendon disease using Dixon-based MRI
Source: Skeletal Radiol. 2024 Dec 11;54(7):1457–68. doi: 10.1007/s00256-024-04845-7 (PMC12078382; doi:10.1007/s00256-024-04845-7)
Supplement: Supplementary file 1 — Supplementary file1 (DOCX 17 KB) [file 256_2024_4845_MOESM1_ESM.docx]

**Supplementary Material**

**Supplementary Table 1:** Inter-reader agreement.

|  | **ICC *** | **Lower 95% CL** | **Upper 95% CL** |
| --- | --- | --- | --- |
| Achilles tendon diameter | 0.926 | 0.887 | 0.951 |
|  | **Cohen’s** κ ***** | **Lower 95% CL** | **Upper 95% CL** |
| Achilles tendon quality | 0.916 | 0.843 | 0.989 |
| Visual grading fatty degeneration MG | 0.929 | 0.862 | 0.996 |
| Visual grading fatty degeneration LG | 0.923 | 0.850 | 0.996 |
| Visual grading fatty degeneration soleus | 0.911 | 0.837 | 0.985 |
| Muscle edema MG | 0.783 | 0.601 | 0.965 |
| Muscle edema LG | 0.839 | 0.663 | 1.000 |
| Muscle edema soleus | 0.754 | 0.613 | 0.895 |
| Kager’s fat pad edema | 0.852 | 0.727 | 0.977 |
| Haglund’s deformity | 0.826 | 0.693 | 0.959 |
| Bone marrow edema in the posterosuperior calcaneus | 0.872 | 0.750 | 0.994 |
| Retrocalcaneal bursitis | 0.796 | 0.671 | 0.921 |

***** The level of agreement was categorized as follows [27]: .0=poor, .01-.20=slight, .21-.40=fair, .41-.60=moderate, .61-.80=substantial, .81-1.00=almost perfect agreement.

*CL, confidence limit; ICC, intraclass correlation coefficient; LG, lateral gastrocnemius (muscle); MG, medial gastrocnemius (muscle).*
